# Lack of evidence for chloramphenicol resistance in Neisseria meningitidis, Africa.

**DOI:** 10.3201/eid0701.010130

**Published:** 2001

**Authors:** M L Tondella, N E Rosenstein, L W Mayer, F C Tenover, S A Stocker, M W Reeves, T Popovic


12. Bisognano C, Vaudaux P, Rohner P, Lew DP, Hooper DC. Induction of fibronectin-binding proteins and increased adhesion of quinolone-resistant *Staphylococcus aureus* by subinhibitory levels of ciprofloxacin. Antimicrob Agents Chemother 2000;44:1428-37.
13. Cars O, Mölstad S, Melander A. Large variation in antibiotic usages between European countries [abstract MoP299]. Clin Microbiol Infect 2000;6(Suppl 1):216.
14. Janknegt R, Oude Lashof A, Gould IM, van der Meer JWM. Antibiotic use in Dutch hospitals 1991-1996. J Antimicrob Chemother 2000;45:251-6.
15. European Antimicrobial Resistance Surveillance System. EARSS Newsletter. Apr 2000 (2). Available from: URL: http://www.earss.rivm.nl/PAGINA/DOC/newslapril2000_arial.pdf

### Lack of Evidence for Chloramphenicol Resistance in *Neisseria meningitidis*, Africa

**To the Editor:** High-level chloramphenicol resistance has been reported in 11 epidemiologically unrelated *Neisseria meningitidis* serogroup B strains in Vietnam and in a single strain in France, all isolated between 1987 and 1996 (1). Resistance was mediated by a chloramphenicol acetyltransferase (Cat) encoded by a *catP* gene homologous to *Clostridium perfringens* transposon Tn*4451*. While used infrequently in industrialized countries, chloramphenicol is often used to treat patients with meningococcal disease in Africa, especially during epidemics, when it frequently becomes the drug of choice because it can be administrated intramuscularly (2).

To evaluate the presence of meningococcal chloramphenicol-resistant isolates in Africa, we assessed the frequency of the *catP* gene in 33 *N. meningitidis* strains of serogroup A from the collection of the Centers for Disease Control and Prevention's Epidemic Investigations Laboratory. The isolates, selected to give the maximum geographic and chronological representation, were collected during 1963 to 1998 from Chad, Egypt, Gambia, Ghana, Niger, Nigeria, South Africa, Tanzania, and Uganda, mostly during outbreaks. Thirteen (39.3%) of the strains were isolated during the 1990s, when chloramphenicol resistance was first described in Vietnam. All isolates were characterized by multilocus enzyme electrophoresis and represented four major electrophoretic subgroups (3,4). Chloramphenicol and penicillin MICs were determined for all isolates, according to the recommendations of the National Committee for Clinical Laboratory Standards, by the broth microdilution method using Mueller-Hinton broth with 5% lysed horse blood incubated in 5% $CO_2$ (5). All isolates were susceptible to both chloramphenicol (MIC <2 µg/mL) and penicillin (MIC <0.06 µg/mL). In addition, we tested all isolates for the presence of *catP* by polymerase chain reaction (PCR) using primers A, B, C, and D (1). Primers A and B, designed from the sequence of *catP,* amplify a 300-bp fragment only in chloramphenicol-resistant isolates. Primers C and D, designed on the basis of meningococcal sequences flanking the Tn*4451*-like insertion, amplify ~1200-bp fragment in resistant isolates and ~200-bp fragment in susceptible strains. Strain LNP13947 (kindly provided by Marc Galimand) was used as a positive control.

The *catP* gene was not detected in 32 of 33 *N. meningitidis* serogroup A strains. One isolate that was negative with primers C and D tested positive with primers A and B (M2786, Nigeria, 1963), which could suggest that *catP* was present but in a different location in the meningococcal genome. However, the chloramphenicol MIC of that strain was 2 µg/mL (susceptible). Repeated attempts to sequence the A/B amplicon were not successful with either primers A and B or another set of primers internal to primers A and B, implying that only a portion of the *catP* gene was present or (even more likely, given the conserved nature of this gene) that the PCR result was a false positive.

Chloramphenicol resistance was first described in meningococcal serogroup B isolates (1), but only serogroup A strains were included in this study since A is the most prevalent serogroup in Africa. (It accounts for most epidemics in Sub-Saharan regions.) Although our small sample size limited the chances of detecting a rare event, the data suggest that chloramphenicol resistance in Africa is relatively infrequent and that chloramphenicol is still an appropriate agent to treat meningococcal disease.

The acquisition of plasmids encoding Cat, which enzymatically inactivate choramphenicol, is the most common mechanism of resistance in gram-positive and gram-negative organisms.

The *catP* gene has been found on various bacterial chromosomes and conjugative plasmids as part of the transposable element Tn*4451*. This transposon is derivative of the Tn*6338* that contains six genes, the largest of which, *TnpX,* is required for the excision of the transposon in both *Escherichia coli* and *C. perfringens* (6). The Tn*4451* derivative that lacked the functional *TnpX* gene was completely stable in both organisms because it had lost mobility as a result of these internal deletions (6). The finding of *catP* in *N. meningitidis* within such a truncated immobile transposon (1) and the possibility of transfer of this type of resistance among highly transformable organisms such as *Neisseria* spp. are of great concern. Were the transposon to become a stable part of the meningococcal genome, it could potentially be easily exchanged. Interspecies recombination between antibiotic-resistant genes of *N. meningitidis* and commensal *Neisseria* spp. has occurred in penicillin- and sulfonamide-resistant meningococci (7-10). A similar occurrence may be possible for *N. meningitidis* chloramphenicol resistance in Africa or other continents where this antibiotic is routinely used for treatment of patients with meningococcal diseases. Studies have not yet demonstrated the clinical significance of chloramphenicol resistance caused by the *catP* gene in meningococci. However, it is possible that, in developing countries, patients whose illness does not respond to antimicrobial agents may not be detected, or their isolates may not be obtained. Screening a selection of isolates for *catP* may allow early detection of chloramphenicol-resistant strains. Since detection of increasing chloramphenicol resistance could change recommendations for antimicrobial-drug therapy, surveillance for antimicrobial-drug resistance should be encouraged.

### Acknowledgment

We gratefully acknowledge Jasmine M. Mohammed for performing chloramphenicol and penicillin MICs.

**Maria-Lucia C. Tondella, Nancy E. Rosenstein, Leonard W. Mayer, Fred C. Tenover, Sheila A. Stocker, Mike W. Reeves, and Tanja Popovic**
Centers for Disease Control and Prevention, Atlanta, Georgia, USA

### References

1. Galimand M, Gerbaud G, Guibourdenche M, Riou JY, Courvalin P. High-level chloramphenicol resistance in *Neisseria meningitidis*. N Engl J Med 1998;339:868-74.
2. World Health Organization. Control of epidemic meningococcal disease. WHO Practical Guidelines. 2nd ed. Geneva: World Health Organization; 1998. p.1-84 (WHO/EMC/BAC/98.3).
3. Selander RK, Caugant DA, Ochman H, Musser JM, Gilmour MN, Whittam TS. Methods of multilocus enzyme electrophoresis for bacterial population genetics and systematics. Appl Environ Microbiol 1986;51:873-84.
4. Caugant DA. Population genetics and molecular epidemiology of *Neisseria meningitidis*. APMIS 1998;106:505-25.
5. National Committee for Clinical Laboratory Standards. Methods for dilution antimicrobial susceptibility tests for bacteria that grow aerobically. 5th edition: approved standard, M7-A5. Wayne, PA: National Committee for Clinical Laboratory Standards; 2000.
6. Bannam TL, Crellin PK, Rood JI. Molecular genetics of the chloramphenicol-resistance transposon Tn4451 from *Clostridium perfringens*: the *TnpX* site-specific recombinase excises a circular transposon molecule. Mol Microbiol 1995; 16:536-51.
7. Spratt BG, Zhang QY, Jones DM, Hutchison A, Brannigan JA, Dowson CG. Recruitment of a penicillin-binding protein gene from *Neisseria flavescens* during the emergence of penicillin resistance in *Neisseria meningitidis.* Proc Natl Acad Sci U S A 1989;86:8988-92.
8. Sáez-Nieto JA, Lujan R, Martinez-Soares JV, Berron S, Vazquez JA, Vinas M, et al. *Neisseria lactamica* and *Neisseria polysaccharea* as possible sources of meningococcal beta-lactam resistance by genetic transformation. Antimicrob Agents Chemother 1990;34:2269-72.
9. Bowler LD, Zhang QY, Riou JY, Spratt BG. Interspecies recombination between the *penA* genes of *Neisseria meningitidis* and commensal *Neisseria* species during the emergence of penicillin resistance in *N. meningitidis*. J Bacteriol 1994;176:333-7.
10. Oppenheim BA. Antibiotic resistance in *Neisseria meningitidis*. Clin Infect Dis 1997;24(suppl 1):S98-S101.

### Iron Loading and Disease Surveillance

**To the Editor:** We read with interest the article by E. D. Weinberg entitled "Iron Loading and Disease Surveillance" (1). Dr. Weinberg proposes routine population screening of iron values by serum ferritin and transferrin saturation tests. Such screening could provide valuable information for epidemiologic, diagnostic,

